# Phosphorylation site topology governs the functional dynamics of arrestin recruitment to GPCRs

**DOI:** 10.21203/rs.3.rs-9071664/v1

**Published:** 2026-04-14

**Authors:** Irene Coin, Timo Müller, Woojin Lee, Ammar Alkara, Asat Baischew, Yasmin Aydin, Jordy Lam, Falko Nagel, Carsten Hoffmann, Stefan Schulz, Andrea Sinz, Vsevolod Katritch

**Affiliations:** Leipzig University; Leipzig University; USC Dornsife; Leipzig University; Martin Luther University Halle-Wittenberg; Indiana University School of Medicine; University of Southern California; 47TM Antibodies GmbH, Jena; Jena University Hospital; Institut für Pharmakologie und Toxikologie, Universitätsklinikum Jena, Friedrich-Schiller-Universität Jena; Center for Structural Mass Spectrometry, Department of Pharmaceutical Chemistry & Bioanalytics, Institute of Pharmacy, Martin Luther University Halle-Wittenberg, Kurt-Mothes-Str. 3, 06120 Halle (Saa; USC

## Abstract

Desensitization and alternative signaling pathways of G protein-coupled receptors (GPCRs) are largely mediated by β-arrestins (arr). While most GPCRs recruit arrestin through phosphorylated C-terminal tails, many lack a canonical tail and instead rely on phosphorylation sites within long third intracellular loops (ICL3). Structural information on such complexes is largely missing, and the molecular details of arrestin engagement remain unknown.

Here, we dissect the interaction between the muscarinic acetylcholine receptor M2 (M_2_R) and β-arrestins directly in living cells using genetically encoded crosslinkers and derive crosslinking-guided atomistic models of the M_2_R-arrestin complex, supported by unbiased molecular dynamics simulations.

Our results show that the M_2_R ICL3 wraps around a positively charged belt on the arrestin N-domain, whereas the receptor core engages the arrestin central crest through a highly dynamic interface. This core contact is essential for efficient arrestin binding and is tuned by the topology of phosphorylation sites, with markedly stronger engagement when the sites reside in a C-terminal tail rather than in ICL3.

Our work provides previously inaccessible structural insight into GPCR-arrestin complexes that rely on ICL3-mediated recruitment and suggests that the topology of the interaction, rather than sequence motifs alone, governs the functional dynamics of arrestin coupling.

## Introduction

Desensitization of G protein coupled receptors in mammals is largely mediated by two ubiquitous b-arrestins (arr2 and arr3, a.k.a. β-arr1 and β-arr2, respectively)^1^, flexible cytosolic proteins that bind activated and phosphorylated GPCRs, block G protein signaling and recruit components of the internalization machinery^2,3,4,5^.

Arrestins feature two concave cup-shaped domains rich in b-sheet structures (N- and C-domain), connected by a central crest including several loops (finger loop, middle loop, lariat loop and gate loop). A few structures of GPCR-arrestin complexes have been solved, mostly representing receptors carrying a highly phosphorylated C-tail, which docks to a positively charged groove of the arrestin N-domain^6^. In most cases, the finger loop inserts into the transmembrane core of the receptor and occupies the same binding site as the C-terminal α-helix of the G protein^7^, as observed in the arr2 complexes with NTS1R^8^, β_1_AR^9^, V_2_R^10^, 5-HT_2B_R^11^, CB_1_R^12^, mGlu3R^13^, and the crosslinking-based PTH1R-arr2 model^14^ (fully engaged conformation). Alternatively, arrestin engages only the phosphorylated receptor C-tail^15,16^, as observed in the structures of the GCGR-arr2 complex^17^ and the β_2_V_2_R-G_s_-arr2 megaplex^18^ (tail conformation). In the ACKR3-arr2 complex^19^, the finger loop of arrestin engages the micelle.

On the other hand, many physiologically relevant GPCRs, including the muscarinic acetylcholine receptors M_1–5_R, the dopamine receptors D_2_R and D_4_R, the adrenergic receptors α_2B_AR and α_2C_AR, and the histamine receptors H_1_R and H_3_R^20^, do not have a phosphorylatable C-tail, but feature instead a long third intracellular loop (ICL3) carrying phosphorylation sites. While there is biochemical evidence that these receptors recruit arrestin via the ICL3^21,22,23,24^, the structural details of this interaction are largely unknown.

The M_2_R is a class A GPCR that mediates the negative chronotropic and inotropic effects of the parasympathetic nervous system on the heart and smooth muscle contraction via coupling to Gi^25,26^, and is a potential therapeutic target for a range of cardiovascular^27^ and neuropsychiatric disorders^28^, and cancer^29^. The human M_2_R features a very short 10-residue C-terminus and a ca. 150 residue-long ICL3 containing at least two phosphorylation clusters, ^286^STSVS^290^ and ^307^TVSTS^311^, the latter being primarily responsible for arrestin recruitment^30^ and M_2_R desensitization^31^. Two cryogenic electron microscopy (cryo-EM) structures depicting arrestin complexes with the M_2_R have been published, both of which fail to depict the natural M_2_R-arrestin interaction. The first study used a chimeric receptor, in which M_2_R was fused to the highly phosphorylated C-tail of the vasopressin receptor (M_2_R–V_2_Rpp–arr2 complex). This chimera stabilizes a fully engaged arr2 conformation in which the receptor is well resolved. As expected, the artificial phospho-tail occupies the same basic groove on arr2 N-domain that is normally engaged by phosphorylated GPCR C-tails. However, the ICL3, which naturally contains the phosphorylation sites responsible for arrestin recruitment, is not resolved^32^. The second arrestin-bound M_2_R structure did use the full-length M_2_R sequence, but resolved only a small 8-residue phosphorylated segment of ICL3 bound in the basic groove of the arrestin N-domain, while the rest of the receptor was too flexible for 3D reconstruction^30^.

It is worth mentioning that all structures of GPCR-arrestin complexes published so far used preactivated forms of arrestins, which do not necessarily give the same interactions as wt arrestins^33^.

Here, we dissect the interaction of the minimally modified full-length M_2_R with arrestin using extensive crosslinking mediated by genetically encoded crosslinkers in intact mammalian cells^34,35^, which provides a comprehensive pairwise proximity map enabling the construction of atomistic models of the M_2_R-arrestin complex. We find that, in living cells, phosphorylated ICL3 wraps around an extended positively charged belt on the arrestin N-domain, while the M_2_R 7TM core engages the arrestin central crest through a highly dynamic yet functionally essential interface. Relocating the phosphorylation sites to a canonical C-terminal tail stabilizes core engagement and enhances arrestin binding, demonstrating that the spatial topology of phosphorylation sites, rather than sequence alone, is a key determinant of receptor–arrestin coupling.

## Results

### Footprint of the M_2_R on arrestin-3

The M_2_R has been shown to recruit both b-arrestins^36^. Using a genetically encoded photo-crosslinker as a proximity probe^34,35^, we mapped the footprint of the M_2_R on the surface of arr3^14,37^. The non-canonical amino acid (ncAA) benzoylphenylalanine (Bpa) was incorporated *via* amber suppression throughout 206 positions of arr3 covering the central crest and the N-domain, a binding interface shared by multiple receptor-arrestin complexes. To improve expression and membrane localization, the receptor was equipped with an N-terminal tag derived from the extracellular domain of the parathyroid hormone 1 receptor (PTH1R) including the DYKDDDDK (FLAG) epitope for immunodetection, whereas the intracellular region was not modified. The PF-M_2_R construct activates Gi and recruits arrestin with EC_50_ values similar to the wild type (Extended Data Fig. 1A-B) and is therefore suitable for crosslinking experiments. Arr3 was equipped with a C-terminal 3xHA-tag, which is well tolerated^14^.

**Figure 1:**
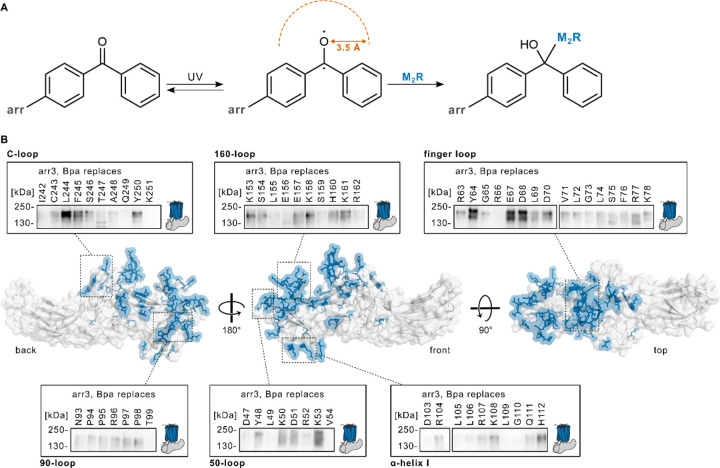
Footprint of the M_2_R on arrestin. **A** UV-induced crosslinking reaction of Bpa-arr with M_2_R yields a covalent M2R-arr complex. **B** Photo-crosslinking hits highlighted in blue on the surface of active arr2 (grey, PDB ID: 6K3F). Insets show a subset of western blots from photo-crosslinking experiments, with the PF-M_2_R-arr3 complex running at 200–250 kDa, detected with an HRP-coupled anti-HA antibody. The full photo-crosslinking mapping is shown in Extended Data Fig. 2.

PF-M_2_R was co-expressed with each Bpa-arr3-3xHA mutant in HEK293 cells depleted of GRK5/6 (HEK293 ΔGRK5/6)^38^. In this way, we aimed at suppressing a nonfunctional phosphorylation background, since only phosphorylation by GRK2 and GRK3 promotes arrestin recruitment to M_2_R^38^. EC_50_ values of arrestin recruitment did not differ between HEK293T and HEK293 ΔGRK5/6 (Extended Data Fig. 1A). Cells were stimulated with acetylcholine (ACh) and photo-crosslinking triggered with UV light. The activated benzophenone captures M_2_R residues coming in its spatial proximity within a radius of ca. 3.5 Å from the diradical oxygen in a quite unspecific fashion (Fig. 1A)^39^. The crosslinked product is detected as a high molecular weight (MW) band on immunoblots^14,37^. Crosslinking occurred only upon receptor activation, with crosslinking bands shifting to lower molecular weights upon deglycosylation, thus confirming their attribution to the M_2_R-arr complex (Extended Data Fig. 1C)^37^. Photo-crosslinking hits are broadly distributed across the concave arr3 surface, with clusters of hits both in the N-domain of arr3 (β-strand I, 50-loop, α-helix I, 160-loop) and the central crest including β-strand VI, the C-loop, the lariat loop and particularly strong crosslinks in the finger loop (Extended Data Fig. 2). We define the set of arr3 residues that captured M_2_R as the footprint of the receptor on the arrestin (Fig. 1B). The numerous photo-crosslinking hits in the central crest of arr3 (finger loop, β-strand VI, and C-loop) together with the numerous hits in the arr3 N-domain (α-helix I, 160-loop and C-loop) are compatible with a fully engaged core conformation of the M_2_R-arr complex.

### Pairwise crosslinking reveals the arrangement of the M_2_R-arr complex

**Figure 2:**
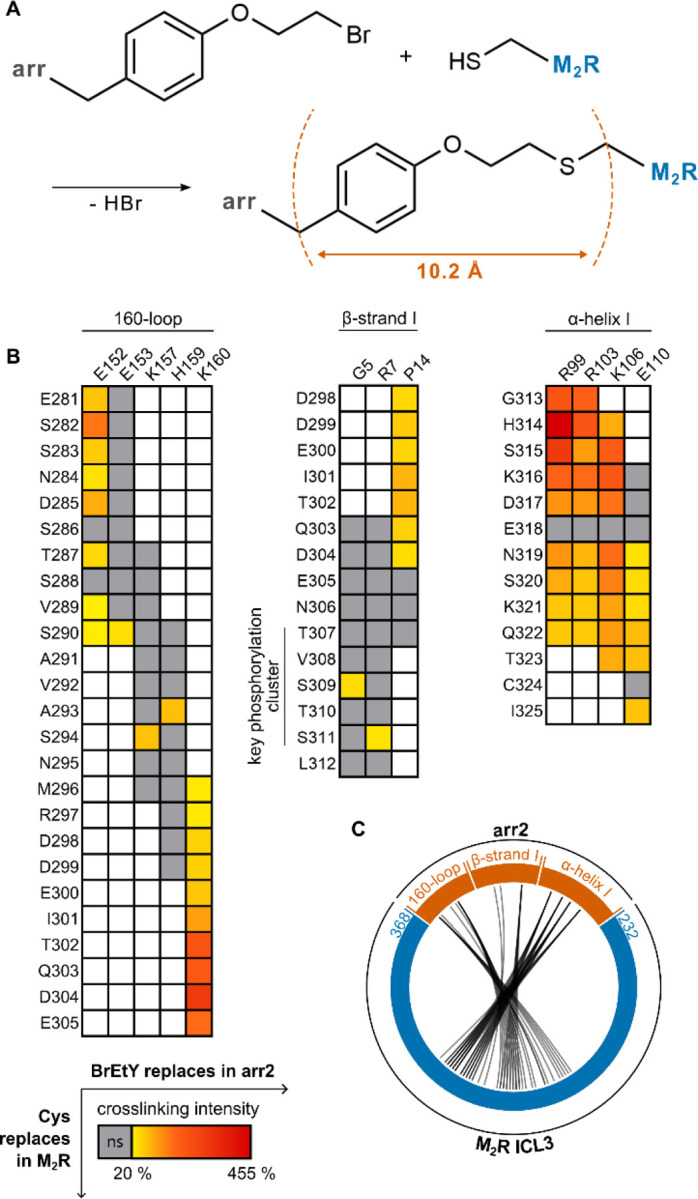
Pairwise crosslinking to identify pairs of proximal residues in the M_2_R and arr2. **A** Proximity-induced crosslinking reaction between BrEtY and a cysteine residue yielding a stable thioether. **B** Crosslinking matrix of the pairwise crosslinking between BrEtY-arr2 mutants (columns) and Cys-M_2_R mutants (rows) of the ICL3. Color-coded according to the crosslinking intensity significantly above background. Gray squares represent non-significant combinations, white squares represent combination not tested. All tested combinations are shown in Supplementary Material 1. **C** Circular contact map of the pairwise crosslinking between arr2 (orange) and) the ICL3 of the M_2_R (blue). Black connecting lines indicate significant crosslinks.

As photo-crosslinking does not provide information about which positions of the receptor are captured by the Bpa-arrestins, we turned to proximity-induced chemical crosslinking between mutually reactive amino acids to identify intermolecular pairs of proximal M_2_R-arrestin residues^34,37^. We applied the irreversible reaction of the mildly electrophilic ncAA BrEtY (O-(2-bromoethyl)-tyrosine)^40^ incorporated into arrestin and Cys residues incorporated into the receptor, which yields an irreversible covalent bond when the alkyl bromide moiety comes near the thiol group (“thiol trapping”, Fig. 2A)^41^ within an estimated Cb-Cb distance of 10.2 Å^40^. Pairwise crosslinking experiments were performed using arr2, because this arrestin is expressed at a much higher level compared to arr3 in HEK293T cells^37^ and yielded in preliminary experiments much clearer and more robust results compared to arr3, as observed also with other GPCRs^37^. Arr2 and arr3 have 78 % sequence identity^42^ and feature high structural similarity^43^. Indeed, we observed that the photo-crosslinking hits detected with arr3 are in general transferable to arr2 (Extended Data Fig. 4A).

Arr2 mutants carrying BrEtY along the photo-crosslinking footprint and in further promising positions selected on the basis of existing structures were expressed in HEK293 ΔGRK5/6 along with PF-M_2_R variants carrying Cys residues throughout the intracellular juxtamembrane region, including the three ICLs and the TM7-hinge region for a total of 1096 combinations. To avoid interference by endogenous intracellular Cys residues in the M_2_R (C124, C274, C324, C337, C439, C443), these were mutated to either serine or alanine, except for C457, which is naturally palmitoylated^44^. These substitutions did not affect Gi activation and arrestin recruitment (Extended Data Fig. 1D-E). To improve crosslinking signals, GRK3 was over-expressed, which did not alter the EC_50_ of arr2 recruitment to the PF-M_2_R (Extended Data Fig. 1F). In a preliminary screen, the occurrence of pairwise crosslinking was assessed using immunoblotting. The intensity of crosslinking hits was then quantified via XL-ELISA, which was performed in at least three biological replicates (Extended Data Fig. 3). The crosslinking intensity was normalized to a positive control, and only crosslinks with a normalized crosslinking intensity above 20 % as well as significantly above background (unpaired t-test, p ≤ 0.02) were considered as hits. The background signal was measured as the crosslinking intensity between BrEtY-arr2 mutants and Cys-depleted PF-M_2_R. In total, 65 significant M_2_R-arr2 crosslinks were detected out of 1096 pairs tested. The crosslinks follow the extended path of the ICL3 wrapping around the N-domain of arr2, including residues upstream of the key phosphorylation cluster proximal to the 160-loop and β-sheet I of arr2 and residues distal of the key phosphorylation cluster proximal to α-helix I of arr2 (Fig. 2B–C), indicating a semicircular arrangement around the N-domain of arr2. As expected, crosslinks observed around the ^307^TVSTS^311^ phosphorylation cluster match with interactions observed in the cryo-EM structure of the ICL3 fragment of the full-length M_2_R in complex with arr2^30^.

Intriguingly, although strong photo-crosslinking hits were found in the central crest of both b-arrestins, we only found two weak pairwise crosslinks between the central crest and the M_2_R: G286BrEtY in the lariat loop of arr2 crosslinked K127C and P132C in the ICL2 of the M_2_R (Extended Data Fig. 4B). Strikingly, arr2 carrying BrEtY at the two most promising positions of arr2, namely Y63 (which yielded the most intense photo-crosslinking signal to M_2_R) and F75 (which is a chemical crosslinking hub to the core of PTH1R^14^), failed to give detectable crosslinking to any of the 88 M_2_R variants carrying Cys along the whole intracellular region of M_2_R, including portions of all transmembrane helices, ICL1, ICL2, helix VIII and the C-terminus (Extended Data Fig. 4C).

### The 7TM core interaction is required for arrestin recruitment

The puzzling lack of strong pairwise crosslinks in the central crest of arr2, even in the finger loop, warranted further investigation. It has been proposed that arrestin binds to the ICL3 of the M_2_R in a hanging conformation that lacks stable interactions with the receptor core^30^. However, we found strong photo-crosslinks to the M_2_R indeed in the arrestin central crest (Fig. 1B, Extended Data Fig. 4A), which usually contacts the GPCR core in the available structures of GPCR-arr complexes.

**Figure 3:**
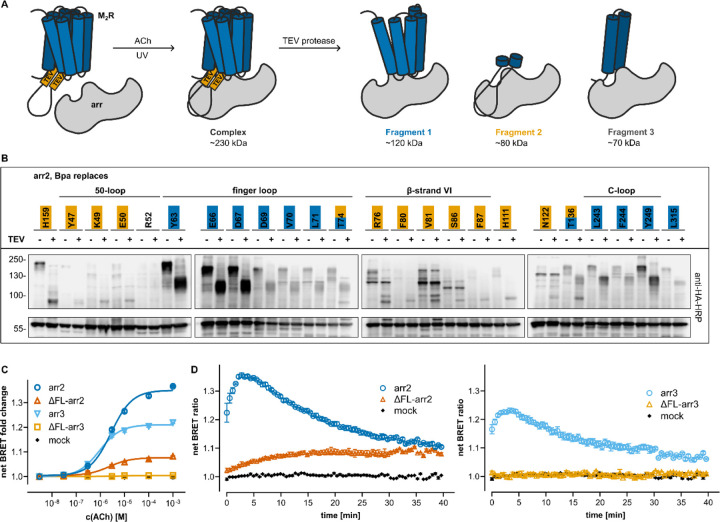
Role of the core interaction in the binding and recruitment of arr2. **A** Schematic representation of the fragments yielded by the cleavage of the crosslinking between Bpa-arr2 and cleavable M_2_R variant. **B** Western blots of cell lysates from photo-crosslinking experiments before and after TEV protease treatment. Crosslinked fragments are indicated by color: dark blue indicates fragment 1, light blue indicates fragment 2. Detected with HRP-coupled anti-HA, anti-FLAG blots are shown in Extended Data Fig. 5D. **C-D** Recruitment of Venus-arr2 (dark blue) and Venus-ΔFL-arr2 (dark orange) as well as Venus-arr3 (light blue) and Venus-ΔFL-arr3 (light orange) to M_2_R-NanoLuc measured via a BRET-based assay. Concentration-response curves (**C**) and kinetic curves at ACh = 100 μM (**D**). Each point represents the arithmetic mean of three independent experiments ± SEM.

In order to determine whether photo-crosslinking hits observed in the central crest capture the M_2_R ICL3 or its core, we generated a cleavable M_2_R variant that carries two tobacco etch virus (TEV) protease cleavage sites at the beginning (between V225 and A226) and at the end (between A373 and K374) of the ICL3 (Extended Data Fig. 5A). The TEV-cleavable M_2_R variant exhibits arrestin recruitment comparable to that of the wt-M_2_R (Extended Data Fig. 5B). TEV-cleavage of the crosslinking product generates three possible receptor fragments crosslinked to the arrestin (Fig. 3A): the N-terminal segment up to the beginning of the ICL3 (~120 kDa, sensitive toward deglycosylation), the ICL3 itself (~80 kDa), or the C-terminal segment starting from the end of the ICL3 (~70 kDa). Thus, the size of the cleaved product reveals the crosslinked region.

A subset of 24 Bpa-arr2 mutants sampling the whole footprint of M_2_R on arr2 was selected and co-expressed with the cleavable M_2_R in HEK293T cells. After activation with ACh and UV irradiation, cell lysates were treated with TEV protease, resolved on SDS-PAGE and immunoblotted (Fig. 3B). The size of the digested products depended on the position of Bpa in the arrestin: arr2 residues in the central crest crosslinked the N-terminal receptor segment, yielding a product featuring the expected shift towards lower molecular weight upon deglycosylation (Extended Data Fig. 5C). In contrast, residues located in the arr2 N-domain crosslinked the ICL3 (Fig. 3B), yielding cleaved crosslinked products that did not respond to PNGaseF treatment (Extended Data Fig. 5C). No crosslinks with the C-terminal M_2_R segment were detected. These results demonstrate that crosslinking hits in the central crest of arrestin capture the M_2_R core.

To establish whether the core interaction is required for arrestin recruitment to the M_2_R, we compared recruitment of wt-arr to that of variants depleted of the finger loop (delY63-K77, i.e. ΔFL-arr2; delY64-K78, i.e. ΔFL-arr3), which are commonly accepted to bind GPCR in the tail-conformation without interacting with the receptor core^16^. We applied a common BRET-based arrestin recruitment assay and titrated the amounts of DNA plasmids to achieve identical expression of wt- and ΔFL-arr2 (Extended Data Fig. 5E). Compared to wt-arr2, ΔFL-arr2 was recruited to the M_2_R to a much lesser extent and with slower kinetics, yielding a maximal BRET signal of ~25 % intensity compared to wt (Fig. 3C) and reaching maximal recruitment about half an hour after stimulation, whereas recruitment of the wild type peaks after circa 3 min (Fig. 3D). The effect was even more striking with arr3, with ΔFL-arr3 showing no detectable recruitment to the M_2_R.

Overall, these results speak for a core-engaged conformation of the M_2_R-arr2 complex in the intact cells, in which the receptor core (i.e., the intracellular ends of TM1, TM2 and TM3, plus ICL1 and ICL2) interacts with the central crest of arrestin, whereas the ICL3 binds to the N-domain. This is despite the difficulty in identifying strong pairwise crosslinks between the M_2_R core and the central crest of arr2 (see Discussion). Moreover, we confirm that this interaction has a functional relevance, as its suppression almost abolishes arrestin recruitment.

### Crosslinking-guided modeling of the M_2_R-arr2 complex

To analyze the chemical crosslinking data in the structural context, we built and optimized a crosslinking-guided, all-atom 3D model of M2R-arr2 complex. The 65 intermolecular residue pairs that were identified as proximal by pairwise crosslinking were translated into soft harmonic restraints between Cb atoms of these residues (Ca for Gly), weighted by the corresponding crosslinking yields. The initial raw models of M_2_R-arr2 were built using Rosetta’s template-based homology modeling pipeline (RosettaCM) guided by these proximity restraints and structural templates for both arrestin binding to the ICL3-phosphorylated fragment (PDB ID 8JAF^30^) and overall assembly of M2R-arr2 complex (PDB ID 6U1N^32^) (Extended Data Fig. 6A). Ten initial models were then refined by extensive energy-based and proximity-restrained conformational sampling in internal coordinates, using ICM-Pro^45^. Consistent with phosphorylation-specific immunoblotting data (Extended Data Fig. 6B) and literature reports^30^, T307, S309, T310, and S311 in the modeled M_2_R’s ICL3 were phosphorylated in the canonical phosphorylation cluster.

**Figure 4:**
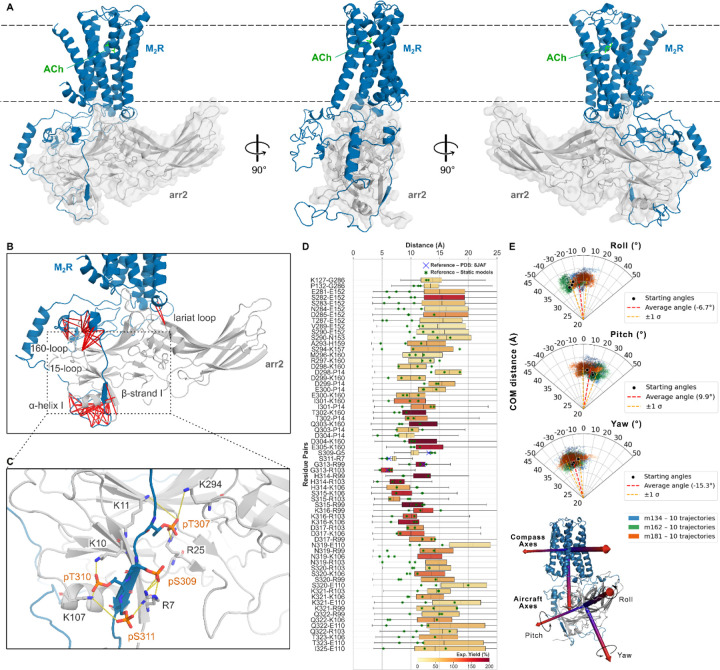
Crosslinking-guided model of the ACh-M_2_R-arr2 complex and its dynamics. **A** Overview of the M_2_R-arr2 complex (model m134). **B** Crosslinking pairs in m134 shown as red lines. **C** Detailed view of the salt bridges between the key phosphorylation cluster ^307^TVSTS^311^ of the M_2_R (labeled in orange) and positively charged residues in the N-domain of arr2 (labeled in black). **D** Distance analysis of the crosslinking pairs during 36 μs aggregate MD simulation time for three starting conformational models (m134, m181, and m162). Ten independent replicates for each model with 1.2 μs long trajectories. Whisker boundaries are the minimum and maximum values; boxes show lower and upper quartiles. The color gradient indicates crosslinking strength. Reference distances from the static models are marked as green asterisks, reference distances from PDB ID 8JAF are marked as blue crosses. **E** Analysis of aircraft axes and arr2–M_2_R COM distance in m134 (blue), m162 (green), m181 (orange) from starting angles (black dots) over 36 μs aggregate simulation time. Red dashed lines represent average angles, yellow dashed lines ± 1σ. Below, the compass and aircraft axes to define roll, pitch and yaw angles are shown.

The refined static models gave a reasonable fit to the crosslinking experimental data, featuring all Cb-Cb distances within 15.0 Å. The best three static models were selected as the representative conformations depicting the fully core-engaged state of the complex (models m134, m181, m162; Fig. 4 A, Extended Data Fig. 6C-D).

Note that some arr2 residues, such as R99, R103, R106, E152 and K160, behave as “crosslinking hubs”, each participating in 8–10 crosslinks with distinct residues of M_2_R (Fig 4B). Obviously, so many interactions cannot be simultaneously satisfied in a single static model due to residue overcrowding around the hub, as discussed in our previous study^14^. Indeed, while almost every crosslinking pair lies within 15 Å, only about half of the pairs satisfy the strict 10.2 Å cutoff, which is the Cb-Cb distance of the Cys-BrEtY adduct (Extended Data Fig. 7A). Because each Cys-BrEtY crosslinking pair was identified in an independent experiment, the simultaneous formation of all Cys-BrEtY adducts must not necessarily occur within a single conformational snapshot, but rather dynamically in the highly flexible complex, as described in the next section.

### Unbiased molecular dynamics of the M_2_R-arr2 complex

To evaluate the dynamic accessibility of the crosslinking pairs and the overall stability of the complex, we carried out unbiased Molecular Dynamics (MD) simulations of three initial M_2_R-arr2 conformational models (m134, m181, m162) embedded in a lipid bilayer, without any crosslinking restraints. For each complex model, ten independent 1.2 microsecond trajectories were generated, yielding 12-microsecond of MD simulations per model, with 36 microseconds in total. At different times during the MD simulations, all the Cb-Cb atoms of experimentally identified crosslinking pairs dynamically came within the 10.2 Å cutoff required for crosslinking (Fig. 4D). Thus, we show that the pairwise crosslinking distances are dynamically accessible in the models without artificial restraints in the simulations.

The conformational stability and flexibility of the whole complex were assessed via backbone RMSD and center-of-mass (COM) distance analyses between M_2_R and arr2 (Extended Data Fig. 7B, Fig. 4E). The M_2_R-arr2 complex shows substantial flexibility, with RMSD reaching ~ 10 Å, and orientation of the receptor and arrestin fluctuating within 30–40° range, with circular standard deviations of 11.8°, 11.0°, and 9.6° for roll, pitch, and yaw, respectively (Fig. 4E). At the same time, M_2_R-arr2 coupling remained consistent in all trajectories, as measured by the distance between receptor and arrestin centers of mass, and without broad reorientation. Notably, important intermolecular contacts, including in and around the key phosphorylation cluster, stay in close proximity during simulations (Fig 4D). Crosslinking pairs further apart from the key phosphorylation cluster vary more broadly in their distances during simulation. Both experimentally observed crosslinks between the M_2_R core and arr2 (K127-M_2_R-G286-arr2 and P132-M_2_R-G286-arr2) persisted throughout the simulations, supporting the validity of the proposed core conformation. Consistent with this, the arr2 finger loop remained deeply inserted between ICL2 and ICL3.

The key phosphorylation cluster ^307^pTVpSpTpS^311^ was carefully analyzed in MD simulations. Frequent salt bridge interactions were observed for pT307 with K11, R25, and K294, pS309 with R7, pT310 with K10 and K107, and pS311 with R7 and K107 (Fig. 4C). Interactions between b-strand I of arr2 and the phosphate groups remained stable throughout the simulations (Extended Data Fig. 8), in agreement with the published cryo-EM data (PDB: 8JAF).

### The topology of the phosphorylation clusters dictates the strength of core interaction

**Figure 5:**
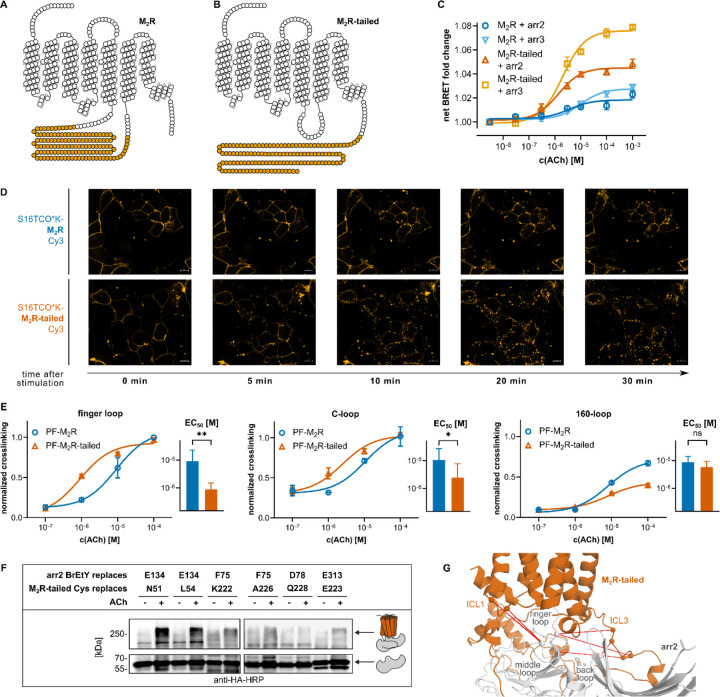
Importance of the location of phosphorylation clusters in ICL3 and C-tail for the core interaction. **A** and **B** Schematic snake representation of the M_2_R (**A**) and the M_2_R-tailed variant (**B**). Residues of the ICL3 that were moved to the C-tail are indicated in orange. **C.** Concentration-response curves of the recruitment of arr2-nLuc to the M_2_R (dark blue) and M_2_R-tailed (dark orange), as well as of arr3-nLuc to the M_2_R (light blue) and M_2_R-tailed (light orange). Arrestin recruitment was measured via a BRET-based bystander assay using YFP-CAAX in three independent biological replicates. Error bars represent the SEM. **D** Internalization of M_2_R and M_2_R-tailed labeled with Cy3-tetrazine at position S16 and observed by wide-field fluorescence microscopy at consecutive time points after stimulation with 10 μM ACh. Scale bar represents 10 μM. **E** Concentration-response curves of the photo-crosslinking between Bpa-arr2 mutants and the PF-M_2_R (blue) as well as the PF-M_2_R-tailed (orange) following stimulation with increasing ACh concentration. Crosslinking intensities were normalized to the crosslinking between Y63Bpa-arr2 and wild-type PF-M_2_R, which was set to 1.0, three independent biological replicates, error bars represent the SEM. Inset bar graphs show the corresponding EC50 values, error bars represent the 95 % CI, stars indicate significance (** ≤ 0.01, * ≤ 0.05, extra-sum-of-squares F test). **F** Western blot of the pairwise crosslinking between BrEtY-arr2 mutants and the PF-M_2_R-tailed without stimulation and following stimulation with ACh. Detected with HRP-coupled anti-HA antibody. **G** Crosslinking pairs between M_2_R-tailed and arr2 illustrated in model m134 (wt-M_2_R-arr2 complex) as red lines. Residues in m134 equivalent to crosslinking residues in the M_2_R-tailed-arr2 complex are highlighted as a sphere placed on Cα. The M_2_R is shown in orange, arr2 is shown in grey.

We further investigated whether the location of the phosphorylation clusters, either in the C-terminus or the ICL3, affects the interaction of arrestin with the receptor core. We reengineered the architecture of M_2_R by moving the ICL3 (134-residue L235–T369 stretch) to the C-terminus. The truncated ICL3 ends were joined to form a short loop, thereby converting the M_2_R into a prototypical GPCR with an average-sized ICL3 and a phosphorylatable C-tail. (Fig. 5 A and B). This “M_2_R-tailed” construct exhibited membrane expression levels similar to those of the wild-type M_2_R (Extended Data Fig. 9A) and induced a similar degree of Gi protein activation (Extended Data Fig. 9B). Moreover, it displayed a phosphorylation pattern largely overlapping with that of the wt receptor, as demonstrated with phosphorylation-specific immunoblotting and mass spectrometry (Extended Data Fig. 9C-D). However, recruitment of both b-arrestins to the M_2_R-tailed construct was markedly greater than to the wild-type M_2_R, as determined by a bystander BRET assay (Fig. 5C). To investigate whether the greater arrestin recruitment reflects into a faster internalization rate of the tailed M_2_R, we equipped the two M_2_R variants with a Cy3 fluorophore at position 15 in the N-terminal domain using bioorthogonal labeling on a genetically encoded chemical anchor, thus leaving the intracellular domains intact^46,47^. Internalization of M_2_R-tailed was already clearly visible 5 minutes after ACh application, whereas the wt-M_2_R required 20 minutes (Fig. 5D).

To investigate whether enhanced arrestin recruitment by the M_2_R-tailed construct stems from a more stable interaction with the receptor core, we compared crosslinking strengths in the core versus other regions as a function of the localization of the phosphorylation cluster. Specifically, we quantified photo-crosslinking intensity obtained at increasing ligand concentration by incorporating Bpa at Y63 (finger loop) and Y249 (C-loop) in the arrestin central crest, which crosslink to the core (Fig. 3B), as well as at E152 in the 160-loop, which crosslinks to the ICL3 (Fig. 2B), using XL-ELISA (Fig. 5E). While both the finger loop and C-loop yielded similar photo-crosslinking with both tailed and wild-type receptor at 100 μM ACh, M_2_R-tailed yielded markedly stronger photo-crosslinking intensity at lower ACh concentrations, with significantly lower EC_50_ values of the corresponding concentration-response curves (p ≤ 0.05, extra sum-of-squares F test). In contrast, the 160-loop mutant showed increasing crosslinking intensity and the same EC_50_ with rising ACh concentrations for both receptor variants. These findings suggest that the arrestin central crest engages the receptor core more efficiently when the phosphorylation clusters are positioned in the C-tail rather than in ICL3.

Indeed, while we could barely find chemical crosslinks between the central crest of arr2 and the core of wt M_2_R, robust chemical crosslinking was readily detected between representative positions in the central crest of arr2 (selected based on our M_2_R-arr2 models) and the core of the M_2_R-tailed (Fig. 5F, Extended Data Fig.10). Hits are visualized in Fig. 5G: ICL1 of PF-M_2_R-tailed crosslinked to the middle loop of arr2, whereas the shortened ICL3 of PF-M_2_R-tailed captured β-strand VI and the back loop.

## Discussion

It is widely accepted that GPCRs recruit arrestins through clusters of phosphorylated residues typically located in their C-tails, which displace an autoregulatory motif of arrestin from a positively charged groove in the N-domain. While subtle differences in local phosphorylation patterns have been proposed to encode distinct intracellular responses (the barcode hypothesis)^48^, whether and how the global spatial distribution of phosphorylation clusters across the GPCR architecture influences arrestin recruitment remains unclear. Here, we apply large-scale ncAA-mediated crosslinking to dissect, directly in intact cells, the interaction between arrestin and the full-length acetylcholine muscarinic M2 receptor, a GPCR that lacks the C tail and recruits arrestin via phosphorylation clusters in its ICL3. We demonstrate that the topology of phosphorylation cluster distribution modulates the strength of the interaction between the arrestin central crest and the GPCR core, thereby controlling both the extent and the kinetics of arrestin recruitment and receptor internalization.

In first place, we show that the M_2_R does require the core interaction to recruit arrestin rapidly and efficiently. Clear evidence that the M_2_R binds arrestin in a fully engaged core conformation is provided by photo-crosslinking. Systematic mapping of the arrestin surface using a Bpa photochemical probe yielded the strongest crosslinks between the arrestin finger loop and the M_2_R core, as unambiguously demonstrated by proteolytic cleavage of photo-crosslinking products. This interaction has a functional relevance, as deletion of the finger loop drastically impaired both the extent and the kinetics of arrestin recruitment to the M_2_R.

On the other hand, results from systematic BrEtY-Cys pairwise crosslinking indicate that, while the interaction between the phosphorylated ICL3 of the M_2_R and the arrestin N-domain is tight and stable, the interaction between the arrestin central crest and the receptor core is considerably more dynamic. Indeed, while we readily identified 65 pairwise contacts between the arrestin N-domain and the M_2_R ICL3, we detected only two BrEtY–Cys crosslinks between the central crest of arrestin and the receptor core. Because radical H-abstractions typically proceed with significantly higher rate constants than S_N_2 reactions under conditions comparable to pairwise crosslinking, photo-crosslinking may more effectively trap highly dynamic interactions^49,50^.

The high dynamic of the core interaction is reflected in our molecular models of the M_2_R-arr2 complex, which were built based on the pairwise crosslinks data. While the fully-engaged conformation remained stable in MD simulations without imposing any restraints, the complex showed relatively high fluctuations, reaching ~ 10 Å RMSD and up to 40° in relative orientation, an overall dynamic that is much more pronounced as compared to what was observed, for instance, for the PTH1R-arr2 complex^14^. Furthermore, the complete set of crosslinking restraints cannot be satisfied simultaneously within a single conformation, whereas all crosslink pairs come within crosslinking distance (≤10.2 Å) during the simulations. This indicates that the identified proximal pairs arise from an ensemble of conformations within the same bound macrostate rather than from a single static structure. Consistently, simulations initiated from multiple starting models yielded largely overlapping distributions of distances and angles. Nonetheless, in line with the experimental observation, crosslinking pairs around the key phosphorylation sites remain very close to each other in the simulations.

In our models, the M_2_R stretch containing the key phosphorylation cluster (^307^pTVpSpTpS^311^) forms an antiparallel b-sheet with b-strand I of arr2 and an extensive salt bridge interaction network between the negative charges and basic residues in the N-domain of arr2, specifically R7, K10, K11, R25, and K107. This is in line not only with the M_2_R-arr2 cryo-EM structure, but also with other published structures of GPCR-arrestin complexes.

Importantly, the crosslinking data enabled reconstruction of a large portion of the M_2_R ICL3, which is unresolved in all available structures. We find that the ICL3 wraps around along a belt of positively charged residues in the arrestin N-domain that engage a distributed series of negatively charged receptor residues through electrostatic interactions. Upstream of the key phosphorylation cluster, a negatively charged motif (^298^DDE^300^) contacts the positively charged distal region of the arrestin N-domain around K160 (the N-edge)^14^. Downstream of the key cluster, D317 and E318 form ionic interactions with the basic residues R103 and K106 in α-helix I of arr2. Negative charges are found upstream of the key phosphorylation sites in many GPCRs^51^, and several receptors also contain additional negative charges downstream of the key cluster; however, this region remains unresolved in available GPCR–arrestin structures. Together, these observations indicate that the distribution of positive charges on arrestin is arranged to accommodate extended stretches of negatively charged residues. We previously observed a similar electrostatic organization in the C-tail of the arrestin-bound PTH1R, and now show that this feature is conserved in receptors whose key phosphorylation sites reside in ICL3.

Altogether, our results suggest that the M_2_R ICL3 binds tightly to the arrestin N-domain, whereas the interaction between the receptor core and the arrestin crest is weaker and less well defined. We show that this reduced core engagement arises from the geometric distribution of phosphorylation sites: relocating ICL3 to the M_2_R C-terminus to mimic the prototypical GPCR architecture generated a “tailed” M_2_R variant that recruited both β-arrestins to a greater extent than the wild-type receptor and internalized more rapidly. Strikingly, whereas BrEtY-Cys crosslinks were barely detectable with wild-type M_2_R, they were readily observed for the core of the tailed receptor, consistent with stronger interactions. As the ICL3 sequence itself was conserved in the tailed receptor and phosphorylation and G protein activation were found identical to the wt M_2_R, the enhanced core engagement is unlikely to reflect changes in intrinsic affinity, but instead results from a more favorable spatial configuration.

In contrast to a canonical C-tail, the ICL3 is tethered at both ends to the transmembrane bundle, potentially imposing geometric and topological constraints on the core engagement. When arrestin is engaged by the phosphorylated receptor tail, it can freely rearrange to bind the receptor core. By contrast, recruitment via a phosphorylated stretch within ICL3 permits stable core engagement only when arrestin is positioned within the loop and adopts the correct orientation. If arrestin initially binds in an alternative topology, it may need to disengage from the phosphorylation site or membrane and subsequently rebind in the proper configuration to access the receptor core. These constraints likely result in a weaker or more dynamic core interaction, which may explain why the receptor core is not resolved in the cryo-EM structures and pairwise crosslinks are difficult to obtain in this region. Previous studies have further shown that the transmembrane core of GPCRs can independently bind and activate arrestins^52^. It is tempting to speculate that the binding mechanism may differ when arrestin approaches receptors phosphorylated in ICL3 rather than in the tail, and that core engagement may not necessarily follow tail binding but could even precede it.

Interestingly, long ICL3s are present in diverse receptor subfamilies^53^ and thus appear to have evolved independently at multiple times. This might suggest advantages of such long ICL3s in specific contexts. For instance, the ICL3 has been proposed to regulate G protein selectivity of some receptors^54^. We now show that the localization of the phosphorylation sites in a long ICL3 has a decisive effect on the interaction with arrestin.

Altogether, our findings provide a structural framework for arrestin recruitment to a GPCR lacking a canonical C-tail and instead harboring its key phosphorylation sites within the ICL3. We show that the M_2_R ICL3 wraps around the arrestin N-domain in a semicircular configuration, while the receptor core simultaneously engages the arrestin central crest through a highly dynamic, transient interface. Moreover, we demonstrate that the strength and stability of this core interaction are dictated by the geometric topology of phosphorylation sites within the receptor. These results redefine current models of arrestin recruitment and indicate that spatial organization, rather than primary sequence alone, governs the geometry and stability of arrestin-GPCR coupling.

## Methods

### Molecular biology

Cloning was performed in *E. coli* DH5α and all enzymes for cloning were purchased from New England Biolabs. PF-M_2_R, M_2_R-tailed, PF-M_2_R-tailed as well as receptor-nLuc, arrestin-nLuc and Venus-arrestin constructs for BRET assays were obtained by overlap extension PCR. Receptor Cys mutants and arr3-3xHA TAG mutants were obtained by high throughput site directed mutagenesis whereas arr2-3xHA TAG mutants were previously obtained^14^. Phusion High Fidelity DNA Polymerase and Q5 High Fidelity DNA Polymerase from New England Biolabs were used for PCR. Mutagenesis primers were designed using AAScan^55^. All oligonucleotides for cloning were purchased from Microsynth and Sanger sequencing was performed by Microsynth Seqlab.

### Cell culture

HEK293T and HEK293 ΔGRK5/6^38^ cells were maintained at 37 °C, 5 % CO_2_ and 95 % humidity in high glucose Dulbecco’s modified Eagle medium (DMEM) with 4.5 g/L glucose, 4 mM glutamine, pyruvate (Thermo Fisher Scientific) and supplemented with fetal bovine serum (Thermo Fisher Scientific), 100 U/mL penicillin and 100 μg/mL streptomycin (Thermo Fisher Scientific) (full DMEM).

### Photo-crosslinking experiments

*p*-Benzoylphenylalanine (Bpa) was purchased from Bachem and stored at room temperature. Before use, it was dissolved in 1 M NaOH and stored at −20 °C.

HEK293T cells were seeded at 450 000 cells per well in a 6-well plate in 2 mL full DMEM whereas HEK293 ΔGRK5/6 cells were seeded at 1 mio cells per well. Cells were incubated for 24 h and then the medium was changed with full DMEM supplemented with 250 μM Bpa. For transfection, PEI (Polysciences) in lactate buffered saline (20 mM sodium lactate at pH 4, 150 mM NaCl) was used at a PEI to DNA ratio of 3:1 (w/w)^56^. Cells were co-transfected with the following plasmids: (1) 900 ng of the arr2/3–3xHA TAG mutant in pcDNA3.1. (2) 300 ng of receptor equipped with a DYKDDDDK (FLAG) epitope tag in pcDNA3.1. (3) 900 ng of BpaRS in pIRE4 (available from ADDGENE #155342)^37^. After 48 h, receptors were stimulated by changing the medium with 100 μM acetylcholine in 1 mL stimulation buffer (PBS supplemented with 0.1 % BSA). Cells were incubated at 37 °C for 30 min and then irradiated with 365 nm UV light (Dymax BlueWave FX-1250 RediCure). The stimulation buffer was discarded, and cells were frozen at −70 °C for 20 min. Then, cells were detached with 1 mL PBS supplemented with 1 μM phenylmethylsulfonyl fluoride (PMSF, Carl Roth) and centrifuged at 2500 xg and 4 °C for 10 min. The pellet was resuspended in 80 μL lysis buffer (20 mM Tris-HCl pH 7.4, 150 mM NaCl, 1 mM EDTA, 10 % (v/v) glycerol, 1 % (v/v) Triton X-100) supplemented with 1x cOmplete protease inhibitor cocktail (Roche) and 2.5 mM dithiothreitol (DTT), incubated on ice for 30 min and regularly vortexed. The samples were centrifuged at 16 000 xg and 4 °C for 10 min and the supernatant was collected.

For TEV cleavage, 0.8 μL of ProTEV Plus (Promega) were added to 10 μL of lysate. The sample was then incubated overnight at 4 °C.

For receptor deglycosylation, 1.1 μL of glycoprotein denaturing buffer (New England Biolabs) were added to 10 μL of lysate. Samples were incubated for 20 min at 37 °C, then 1.4 μL of GlycoBuffer2 (New England Biolabs), 1.4 μL of NP-40 and 0.2 μL of PNGase F (New England Biolabs) were added. Samples were again incubated for 1 h at 37 °C.

### Pairwise crosslinking experiments

Bromoethyltyrosine (BrEtY) was previously synthesized^14^ and stored dry at room temperature. Immediately before the experiment, it was dissolved in DMSO (Carl Roth).

HEK293 ΔGRK5/6 cells were seeded at 1 mio cells per well in a 6-well plate in 2 mL full DMEM. Cells were incubated for 24 h, the medium was changed with full DMEM supplemented with 250 μM BrEtY and cells were transfected with the following plasmids as described above: (1) 900 ng of the arr2-3xHA TAG mutant in pcDNA3.1 (In case of G286BrEtY-arr2: C140S-arr2-3xHA TAG mutant). (2) 300 ng of receptor Cys mutant equipped with a DYKDDDDK (FLAG) epitope tag in pcDNA3.1. (3) 300 ng of GRK3 in pcDNA3. (4) 900 ng of MmXYRS/4xM15-tRNA in pNEU (available from ADDGENE #155343)^37^. After 48 h, receptors were stimulated by changing the medium with 100 μM acetylcholine in 1 mL stimulation buffer (PBS supplemented with 0.1 % BSA). Cells were incubated at 37 °C for 1.5 h, stimulation buffer was aspirated and cells were frozen at −70 °C for 20 min. Cells were lysed as described above.

### SDS-PAGE and western blotting

7 μL of SDS sample buffer (62.5 mM Tris-HCl pH 6.8, 2 % (w/v) SDS, 10 % (v/v) glycerol, 0.05 mg/mL bromophenol blue, 150 mM DTT) were added to 5 μL of lysate and samples were incubated at 37 °C for 30 min. Afterwards, samples were resolved on 8 % Tris-glycine polyacrylamide gels and transferred to Immobilon-FL PVDF membranes (pore size 0.45 μm, Millipore). For 1 h, the membranes were blocked in 5 % non-fat dry milk (NFDM, Carl Roth) in TBS-T (20 mM Tris-HCl at pH 7.4, 150 mM NaCl, 0.1% (v/v) Tween-20) at room temperature. The membranes were incubated under gentle agitation in 1:5000 antibody dilutions (rat 3F10 anti-HA-HRP, Roche and mouse M2 anti-FLAG-HRP, Sigma-Aldrich) in 2 % NFDM at 4 °C overnight. On the next day, the membranes were washed in TBS-T for 3x 10 min. The membranes were then covered with enhanced chemiluminescence reagent (1 mL of 250 mg/mL luminol in 0.1 M Tris at pH 8.6, 100 μL of 1100 mg/mL *p*-coumaric acid in DMSO, 0.3 μL of 30 % H_2_O_2_) and imaged for 5 min in an Li-Cor Odyssey M imager.

### XL-ELISA

Lysates were adjusted to 360 μL with lysis buffer supplemented with 1x protease inhibitor cocktail and 2.5 mM DTT, and 100 μL of lysate per well were loaded onto clear anti-DYKDDDDK coated 96-well plates (GenScript) in three technical replicates. The plates were incubated overnight under gentle agitation at 4 °C. Then, the plates were washed with 3x 300 μL high salt wash buffer (1 M NaCl, 5 % (v/v) Tween-20 in PBS) and 1x 300 μL PBS-T (0.05 % (v/v) Tween-20 in PBS). 100 μL of 1:1000 anti-HA-HRP dilution in 1 % NFDM in PBS per well were added to the plates before incubation under gentle agitation for 1.5 h at room temperature. After washing with 3x 300 μL PBS-T, 100 μL of 6.2 mM *o*-phenylenediamine (OPD) in substrate buffer (50 mM citrate, 50 mM Na_3_PO_4_, pH 5.0) supplemented with 0.08 % of H_2_O_2_ per well were added and plates were incubated for 20 min in the dark. 20 μL of 1 M HCl per well were added to stop the color reaction. Absorption at 492 nm was measured in an Agilent BioTek Synergy Neo2 plate reader.

XL-ELISA were performed in at least three independent experiments, and data are presented as mean ± SD (R7-BrEtY-, G286BrEtY-arr2: 4 independent experiments). Significance above background of pairwise crosslinking hits was tested using an unpaired two-sided t-test (with Holm-Šidák correction for multiple comparisons). All calculations were made with GraphPad Prism 10 (Graphpad Software Inc.).

### Phosphorylation-specific immunoblotting

HEK293 ΔGRK5/6 cells were seeded at 5.9 mio cells per dish in 10 cm dishes. After 24 h, the medium was changed to fresh full DMEM and cells were transfected as described above with the following plasmids: (1) 1.77 μg of PF-M_2_R or FLAG-M_2_R in pcDNA3.1. (2) 10.03 μg of empty pcDNA3.1. Cells were incubated for 48 h, then the medium was exchanged with 100 μM ACh in 5 mL of stimulation buffer. Cells were frozen at −70 °C for 20 min, detached and pelleted as described above. Lysis was carried out with 600 μL lysis buffer supplemented with 1x cOmplete protease inhibitor cocktail (Roche) and 1x PhosSTOP phosphatase inhibitor (Roche) as described above.

Immunoprecipitation was performed using anti-DYKDDDDK (L5) affinity gel (BioLegend). Beads were incubated with the lysate under gentle agitation overnight at 4 °C, washed with 3x 1000 μL high salt wash buffer and eluted with SDS sample buffer sufficient for 4 samples. Samples were resolved on polyacrylamide gels and transferred to PVDF membranes as described above. After blocking in 5 % NFDM for 1 h at room temperature, the membranes were incubated overnight under gentle agitation in 1:1000 primary antibody dilutions (rabbit anti-M_2_R, anti-pT271-M_2_R, anti-pS282/pS283-M_2_R, anti-pS286/pT287/pS288-M_2_R, anti-pT307/pS309-M_2_R, anti pT310/pS311-M_2_R, 7TM Antibodies) at 4 °C. The membranes were washed with TBST-T for 3x 10 min, incubated for 1 h under gentle agitation in 1:10000 secondary antibody dilutions (goat anti-rabbit IRDye 680RD, Li-Cor) at room temperature and again washed with TBS-T for 3×10 min. The membranes were imaged in a Li-Cor Odyssey M imager.

### Arrestin recruitment experiments

HEK293T were seeded at 450 000 cells per well in a 6-well plate in 2 mL full DMEM and HEK293 ΔGRK5/6 cells were seeded at 1 mio cells per well. After 24 h, cells were transfected as described above.

For direct BRET experiments, cells were transfected with the following plasmids: (1) 400 ng of receptor-nanoLuc in pcDNA3.1. (2) 660 ng of Venus-arrestin in pcDNA3.1. (3) 940 ng of empty pcDNA3.1. Alternatively, cells were co-transfected with 300 ng of GRK3 in pcDNA3 and empty pcDNA3.1 was reduced to 640 ng.

For direct BRET experiments involving ΔFL-arrestin, plasmid amounts used for transfection were adjusted according to the relative expression level. Therefore, cells were transfected with either (1) 400 ng of receptor-nanoLuc in pcDNA3.1. (2) 460 ng of Venus-arrestin in pcDNA3.1. (3) 1120 ng of empty pcDNA3.1 or (1) 400 ng of receptor-nanoLuc in pcDNA3.1. (2) 920 ng of Venus-ΔFL-arrestin in pcDNA3.1. (3) 640 ng of empty pcDNA3.1.

For bystander BRET experiments, cells were transfected with the following plasmids: (1) 750 ng of receptor in pcDNA3.1. (2) 150 ng of arr-nLuc in pcDNA3.1. (3) 1500 ng of CAAX-YFP in pcDNA3. (4) 600 ng of empty pcDNA3.1.

Cells were incubated for 24 h and resuspended in 4 mL of full DMEM. 100 μL per well were transferred to a white flat bottom 96-well plate (Greiner) coated with poly-D-lysine (PDL, Merck) in four technical replicates. After another 24 h of incubation, the plates were washed with 2x 90 μL of Hank’s balanced salt solution (HBSS, Thermo Fisher Scientific) supplemented with 25 mM 4-(2-hydroxyethyl)-1-piperazineethanesulfonic acid (HEPES, Carl Roth) per well. 90 μL of h-coelenterazine solution (2.28 μg/mL h-coelenterazine in HBSS supplemented with 25 mM HEPES, Nanolight Technologies) per well were added and baseline signals were measured at 480 nm and 530 nm in an Agilent BioTek Synergy Neo2 plate reader. Then, 10 μL of ligand solution per well were added and signals were again measured at 480 nm and 530 nm. Calculations and non-linear regression were conducted in GraphPad Prism 10 (Graphpad Software Inc.). Mean, SEM, curves and EC_50_ values were calculated from three independent experiments. EC_50_ values were compared using a two-sided extra sum-of-squares F test (if necessary, multiple comparisons were accounted for using the Bonferroni correction).

### G protein dissociation experiments

HEK293T were seeded at 450 000 cells per well in a 6-well plate in 2 mL full DMEM and HEK293 ΔGRK5/6 cells were seeded at 1 mio cells per well. After 24 h, cells were transfected as described above. Cells were transfected with the following plasmids: (1) 200 ng of Gαi_1_-LgBiT in pcDNA3.1. (2) 1000 ng of Gβ_1_ in pcDNA3.1. (3) 1000 ng of SmBiT-Gγ_1_ in pCAGGS. (4) 400 ng of receptor in pcDNA3.1. (5) 200 ng of RIC8A in pCAGGS^57^. After 24 h, cells were transferred into white 96-well plates as described above. Cells were incubated for 24 h, washed and h-coelenterazine solution was added as described above. Baseline bioluminescence was measured at 480 nm in an Agilent BioTek Synergy Neo2 plate reader. Then, 10 μL of ligand solution per well were added and bioluminescence was again measured at 480 nm. Calculations and non-linear regression were conducted in GraphPad Prism 10 (Graphpad Software Inc.). Mean, SEM, curves and EC_50_ values were calculated from three independent experiments. EC_50_ values were compared using a two-sided extra sum-of-squares F test.

### Measurement of relative expression levels of arrestin

HEK293 ΔGRK5/6 cells were seeded at 1 mio cells per well in a 6-well plate. Cells were incubated for 24 h and transfected with the following plasmids: (1) Venus-arr2 or Venus-ΔFL-arr2 in pcDNA3.1. (2) CFP in pcDNA3.1. (3) Empty pcDNA3.1 to a total of 2000 ng. Cells were transferred to black 96-well plates coated with PDL as described above. After 24 h, cells were washed with 2x 100 μL of HBSS supplemented with 25 mM HEPES per well. Then, 100 μL of HBSS supplemented with 25 mM HEPES per well were added and fluorescence was measured at 481 nm and 532 nm. Measurements of relative expression levels were performed in three independent experiments.

### Surface ELISA

HEK293 ΔGRK5/6 cells were seeded at 90 000 cells per well in a PDL-coated 48-well plate. After 24 h, cells were transfected with the following plasmids: (1) 20 ng FLAG-receptor in pcDNA3.1. (2) 180 ng of empty pcDNA3.1. After another 24 h, cells were fixed with 100 μL 4 % paraformaldehyde in PBS for 10 min. Cells were washed with 3x 500 μL PBS and then blocked with 500 μL full DMEM for 1 h at 37 °C. The medium was removed and 1:1000 mouse M2 anti-FLAG-HRP (Sigma-Aldrich) were added, cells were incubated for 1.5 h at 37 °C and then washed with 3x 500 μL PBS. 250 μL of 6.2 mM *o*-phenylenediamine (OPD) in substrate buffer (50 mM citrate, 50 mM Na_3_PO_4_, pH 5.0) supplemented with 0.08 % of H_2_O_2_ per well were added and plates were incubated for 30 min in the dark. The reaction was stopped with 50 μL 1 M HCl, 200 μL supernatant were transferred to a transparent 96-well plate and absorption at 492 nm was measured on an Agilent Synergy BioTek Neo2 plate reader. Surface ELISA were performed in at least three independent experiments, and data are presented as mean ± SD. Surface expression levels were compared using an unpaired two-sided t test. Calculations were performed in GraphPad Prism 10 (Graphpad Software Inc.).

### Mass spectrometry

HEK293 ΔGRK5/6 cells were seeded at 15.1 mio cells per dish in 2x 15 cm dishes. After 24 h, the medium was changed to fresh full DMEM and cells were transfected as described above with the following plasmids: (1) 4.5 μg of FLAG-M_2_R/FLAG-M_2_R-tailed in pcDNA3.1. (2) 25.5 μg of empty pcDNA3.1. Cells were incubated for 48 h, then the medium was exchanged with 100 μM ACh in 15 mL of stimulation buffer. Cells were frozen at −70 °C for 20 min, detached and pelleted as described above. Lysis was carried out with 2 mL MS lysis buffer (1 % DDM, 1 % Triton X-100, 150 mM NaCl, 100 mM TRIS, 160 U DNAse) supplemented with 2x cOmplete protease inhibitor cocktail (Roche) and 2x PhosSTOP phosphatase inhibitor (Roche) as described above. Samples were enriched by IP using anti-FLAG (M2) magnetic beads (Sigma). Samples were eluted with 0.1 M glycine pH 3.0, denatured in 10 % SDS and 100 mM Tris-HCl (pH 7.5), and subjected to S-Trap enrichment (Protifi). Proteins were reduced with 5 mM tris(2-carboxyethyl)phosphine for 15 min at 55°C, alkylated with 20 mM iodoacetamide, and enzymatically digested with trypsin overnight and samples were then lyophilized.

Peptide mixtures were reconstituted in an aqueous solution of 3% (v/v) ACN and 0.05% (v/v) TFA prior to LC-TIMS-MS/MS analysis on an UltiMate 3000 RSLC nano-HPLC system (Thermo Fisher Scientific) coupled to a timsTOF Ultra 2 mass spectrometer (Bruker Daltonics). Peptides were trapped and desalted on a C18 precolumn (Acclaim PepMap 100, 300 μm × 5 mm, 5 μm, 100 Å, Thermo Fisher Scientific) with aqueous 0.1 % (v/v) TFA at a flow rate of 30 μL/min (precolumn temperature 50°C). Using a flow of 300 nL/min, peptide mixtures were then eluted and separated on a self-packed Picofrit C18 column, 75 μm ID × 40 cm, Tip ID 15 μm (New Objective), packed with Reprosil-Pur 120 C18-AQ, 3 μm, 120 Å material (Dr. Maisch GmbH). Linear gradients from 3% to 40% B over 90 min, 50% to 85% B (over 5 min) and 85% B (5 min) were used; solvent A: water containing 0.1% (v/v) formic acid and solvent B: acetonitrile containing 0.1% (v/v) formic acid, the separation column was kept at 40 °C. Data collection was performed in data-dependent acquisition (DDA) modes using parallel accumulation-serial fragmentation (PASEF) with 166 ms ramps for ion accumulation and separation. The mobility-dependent collision energy ramping settings were 59 eV at an inversed reduced mobility (1/k_0_) of 1.6 V s–1 cm–2 and 20 eV at 0.6 V s–1 cm–2. Collision energies were interpolated linearly between the two 1/k_0_ values and were kept constant above or below. The target intensity per individual PASEF precursor was set to 20,000 with an intensity threshold of 500. 10 PASEF MS/MS scans were triggered per acquisition cycle corresponding to a cycle time of 1.89 s. Precursor ions in a m/z range between 100 and 1,700 with charge states between 0–5 were selected for fragmentation. Active exclusion was enabled for 0.5 min (mass width 0.015 Th, 1/K_0_ width 0.100 V•s/cm2).

Phosphopeptides were identified using PEAKS Studio (Version 10.6) with identification settings as follows: trypsin, specific, with a maximum of three missed cleavages, precursor and fragment ion error tolerance of 10 ppm and 0.02 Da; carbamidomethylation (+57.02) of cysteine as fixed modification and oxidation (+15.99) of methionine, and phosphorylation (+79.97) of serine, threonine or tyrosine as variable modification. A maximum of three modifications were allowed per peptide. The following parameters were used to identify phosphorylated peptides: maximum AScore^58^, e.g. 1000, peptides found as unphosphorylated species, and different peptides found with the same phosphorylation site. A biological triplicate was measured for both receptor species. All data have been deposited to ProteomeXchange with identifier PXD071318 (Username: reviewer_pxd071318@ebi.ac.uk, password: dlwLWBBaPw2K).

### Wide-field fluorescence microscopy

Cells were seeded at 70 000 cells per well in 4-well μ-slides (ibidi) coated with PDL. After 24 h, *trans*-cyclooct-2-ene lysine (TCO*K) was added to each well to a final concentration of 250 μM. After another 1 h, cells were transfected using Lipofectamine 2000 (Thermo Fisher Scientific) with the following plasmids: (1) 500 ng of S16TAG-M_2_R/S16TAG-M_2_R-tailed in pcDNA3.1. (2) 300 ng of MaPylRS^AF^/4xMa-tRNA in pIRE4. Cells were incubated for 24 h. One hour prior to labeling, the medium was exchanged with full DMEM. Cy3-tetrazine (Jena Bioscience) was dissolved in DMSO, diluted in full DMEM to 1.5 μM and applied to the cells for 5 min at 37°C^59^. Cells were washed with 500 μL FluoroBrite DMEM supplemented with GlutaMAX (Thermo Fisher Scientific) before another 500 μL FluoroBrite DMEM were added.

Baseline images were acquired immediately before addition of the ligand (t = 0 min). ACh was added at a concentration of 10 μM and images were acquired 5, 10, 20 and 30 min after stimulation on an Axio Observer.Z1/7 microscope (Zeiss) using the following filter settings (bandpass/bandstop, nm): 545–565/575 (excitation) and 579–604 (emission). Image acquisition was controlled using ZEISS ZEN 3.4 light.

### Construction and optimization of static models

The raw M_2_R-arr2 complexes were modeled from the Robetta server with comparative modeling and harmonic constraints (10 ± 4 Å)^60,61,62,63^. Initial M_2_R-arr2 models were assembled in a two-stage protocol. First, a partial segment of the ICL3 (E281–I325) containing the key phosphorylation cluster segment bound to arr2 was rebuilt in Robetta using the cryo-EM structure with PDB ID 8JAF^30^ as template; arr2 loop regions that are absent in 8JAF were remodeled using the arrestin structure with PDB ID 6U1N^32^, which preserved the native 8JAF backbone while completing the missing segments. Second, the partial ICL3/arr2 models were aligned in 6U1N to model M_2_R-arr2 complexes. Robetta was then used to rebuild the missing ICL3 segments and to produce native sequence M_2_R-arr2 starting models for subsequent refinement. T307, S309, T310, and S311 in the modeled M_2_R’s ICL3 were phosphorylated using ICM-Pro^45^.

The ten M_2_R-arr2 starting models (Extended Data Fig. 6B) were imported into ICM-Pro v.3.9.4 (Molsoft LLC) for an extensive energy-based, crosslinking-guided Biased Probability Monte Carlo (BPMC) sampling combined with local gradient minimization in internal coordinates^45^ for ten independent trials, following the procedure described in the previous report^14^. During sampling, arr2 was allowed to reorient, and flexible receptor and arrestin loop conformations were sampled, while the receptor’s transmembrane bundle and arrestin’s β-sheet core conformations were restrained and periodically relaxed by gradient minimization. Crosslinking yields B_ij_ were introduced to the ICM conformational optimization protocol as weights in a soft flat-bottomed harmonic potential energy penalty (E_penalty_) on distance d_ij_ between Cb-Cb atoms of the corresponding crosslink pair in Eq. (1), where the borders of the flat bottom are set at 4.0 Å and at 10.2 Å.


(1) $$E_{\text{penalty}}=\sqrt{B_{ij}}\left[\text{max}{\left(d_{ij}-10.2,0\right)}^{2}+\text{max}{\left(4.0-d_{ij},0\right)}^{2}\right]$$

The lower bound 4.0 Å was selected to penalize atomic clash, while the upper bound was set as 10.2 Å, which is the maximal Cb-Cb distance in the BrEtY-Cys adduct. The entire model underwent energy-based optimization (Metropolis Monte Carlo and gradient descent) with variable strength of harmonic restraints using the Biased Probability Monte Carlo approach until convergence^45^.

### Molecular dynamics (MD) simulations

The optimized M_2_R-arr2 static models were uploaded to the CHARMM-GUI webserver to generate input files for molecular dynamics (MD) simulations^64,65,66^. The structures were embedded in a lipid bilayer composed of 1,2-dipalmitoylphosphatidylcholine (DPPC), dioleoyl phosphatidylcholine (DOPC), and cholesterol (CHL1) at a ratio of DPPC:DOPC:CHL1 = 0.55:0.15:0.30, referencing the setup commonly used in GPCR simulations^67^. The system was solvated with TIP3P water. All ligands were parameterized accordingly using the Ligand Reader & Modeler tool in the CHARMM-GUI interface (v.3.7). All MD simulations were conducted with GROMACS (v.2024.3)^68,69,70^ simulation engine under CHARMM36 force-field parameters and topologies^64^. Ten independent trajectories were simulated starting from the assembled system. All systems were equilibrated for 60 ns after initial energy minimization, followed by production runs of 1200 ns under NVT ensemble with V-rescale thermostat at 310 K. The simulations were performed on a GPU cluster at the Center for Advanced Research Computing (CARC) of the University of Southern California. MD trajectories were analyzed using MDTraj^71^ and MDAnalysis^72^ packages. Structural figures were prepared using ICM-Pro v.3.9.4 and PyMOL^73^.

We quantified the relative orientations between M_2_R and arr2 using Euler angles, as shown in Figure 4E^74^. Principal components (PCs) of arr2 coordinates (Ca only) were calculated on the fly and sorted in descending order by their eigenvalues. In arr2, PC1, PC2, and PC3 define the longitudinal, vertical, and transverse axes, respectively; the direction of the longitudinal axis is fixed by defining nose residues on the arrestin (residues K232–I233, V325–K326, A344–V345), and the vertical axis is set to point away from the membrane. Similarly, PCs of the receptor were used to define the north, east, and down axes, where the longitudinal axis of the receptor is fixed to nose residues on helix IV (residues W148–S151). The vertical axis of the receptor points to the intracellular side of the membrane normal to the lipid bilayer. The pitch, roll, and yaw angles are the Euler angles that align arrestin’s principal axes X_Arr_ to that of the receptor X_M2R_. For each trajectory frame, we can solve X_M2R_ = RX_Arr_ for the rotation matrix R. The directions of these right-handed axes are fixed as described above. The Euler angles are then obtained from R by calculating pitch b, roll g, yaw a angles with equations (2), (3), (4), respectively.


(2) $$\beta ≕\text{arctan}2\left(-R_{31},\sqrt{R_{11}^{2}+R_{21}^{2}}\right)$$

(3) $$\gamma ≕\text{arctan}2\left(\frac{R_{32}}{\text{cos}(\beta )},\frac{R_{33}}{\text{cos}(\beta )}\right)$$

(4) $$\alpha ≕\text{arctan}2\left(\frac{R_{21}}{\text{cos}(\beta )},\frac{R_{11}}{\text{cos}(\beta )}\right)$$

## Supplementary Material

This is a list of supplementary files associated with this preprint. Click to download.

- SupplementaryMaterial1.xlsx
- M2RExtendedData18.docx

## Data Availability

The authors declare that the data supporting the findings of this study are available within the paper and its supplementary information files. The M_2_R-arr2 models generated in this study have been deposited in the PDB-IHM under accession code 9AAI. Molecular dynamics data have been deposited in Zenodo (10.5281/zenodo.18751164 & 10.5281/zenodo.18750118). Mass spectrometry data have been deposited to ProteomeXchange with identifier PXD071318 (Username: reviewer_pxd071318@ebi.ac.uk, password: dlwLWBBaPw2K). Source data are provided with this paper. Zenodo is currently under embargo. Files are accessible through these links: https://zenodo.org/records/18750120?token=eyJhbGciOiJIUzUxMiJ9.eyJpZCI6IjQwYTc5NzNlLTNlYjEtNDA4YS04MDgwLWNhNWE5OGJjZDcyZCIsImRhdGEiOnt9LCJyYW5kb20iOiJjNDYyNjEyZjgxYWX89Ps0-rok2BCN8W_vBTasSv8srgsXJGsQ6657 https://zenodo.org/records/18751165?token=eyJhbGciOiJIUzUxMiJ9.eyJpZCI6IjhmM2M5MjNhLWMwMzgtNDI0NS1hODcxLTU4YzBiMmI1YjdlOSIsImRhdGEiOnt9LCJyYW5kb20iOiJhMTMyNDVhOW
